# Antennal transcriptome sequencing and identification of candidate chemoreceptor proteins from an invasive pest, the American palm weevil, *Rhynchophorus palmarum*

**DOI:** 10.1038/s41598-021-87348-y

**Published:** 2021-04-15

**Authors:** Francisco Gonzalez, Jibin Johny, William B. Walker, Qingtian Guan, Sara Mfarrej, Jernej Jakše, Nicolas Montagné, Emmanuelle Jacquin-Joly, Abdulaziz S. Alqarni, Mohammed Ali Al-Saleh, Arnab Pain, Binu Antony

**Affiliations:** 1 Chair of Date Palm Research, Center for Chemical Ecology and Functional Genomics, Department of Plant Protection, College of Food and Agricultural Sciences, King Saud University, Riyadh, 11451 Saudi Arabia; 2 Department of Research and Development, ChemTica Internacional S.A., Santo Domingo, Heredia Costa Rica; 3 Department To Plant Protection Biology, Swedish University of Agricultural Sciences, Alnarp, Sweden; 4 Biotechnical Faculty, Agronomy Department, University of Ljubljana, 1000 Ljubljana, Slovenia; 5 INRAE, Sorbonne Université, CNRS, IRD, UPEC, Université de Paris, Institute of Ecology and Environmental Sciences of Paris, iEES-Paris, 78000 Versailles, France; 6 BESE Division, King Abdullah University of Science and Technology (KAUST), Thuwal, 23955-6900 Jeddah Saudi Arabia

**Keywords:** Ecology, Evolution, Genetics, Molecular biology, Zoology

## Abstract

For decades, the American palm weevil (APW), *Rhynchophorus palmarum*, has been a threat to coconut and oil palm production in the Americas. It has recently spread towards North America, endangering ornamental palms, and the expanding date palm production. Its behavior presents several parallelisms with a closely related species, *R. ferrugineus*, the red palm weevil (RPW), which is the biggest threat to palms in Asia and Europe. For both species, semiochemicals have been used for management. However, their control is far from complete. We generated an adult antennal transcriptome from APW and annotated chemosensory related gene families to obtain a better understanding of these species' olfaction mechanism. We identified unigenes encoding 37 odorant-binding proteins (OBPs), ten chemosensory proteins (CSPs), four sensory neuron membrane proteins (SNMPs), seven gustatory receptors (GRs), 63 odorant receptors (ORs), and 28 ionotropic receptors (IRs). Noticeably, we find out the *R. ferrugineus* pheromone-binding protein and pheromone receptor orthologs from *R. palmarum*. Candidate genes identified and annotated in this study allow us to compare these palm weevils' chemosensory gene sets. Most importantly, this study provides the foundation for functional studies that could materialize as novel pest management strategies.

## Introduction

Insects live embedded in a chemical environment, in which their survival depends on the proper understanding of those chemical signals. Food, sex, predators, pathogens, and sites to inhabit and oviposit are sources of characteristic chemical signatures that insects must perceive and react to^1,2^. The ability to discriminate among many chemical stimuli and use this information is a feature given by a robust and sophisticated chemosensory system based on a set of specialized proteins^3,4^. These proteins' function spans from transporting odor molecules from the environment to cascading the signal they convey to higher brain levels^2^. Odorants are intercepted by porous hair-like structures on the antennae and palps' surface, called olfactory sensilla. Once the molecules penetrate the sensillum, small soluble proteins present in the lymph may facilitate their movement until they reach the dendritic membrane of olfactory sensory neurons (OSNs). These proteins are called odorant-binding proteins (OBPs) and chemosensory proteins (CSPs)^5^. At the OSN membrane, odor molecules interact in a lock-key fashion with specialized seven-transmembrane domain proteins known as odorant receptor proteins (ORs). Insect ORs form heteromers constituted of two types of proteins that act as ligand-gated ion channels in OSNs. One of these proteins binds to the semiochemicals and determines OSN response specificity and sensitivity.

In contrast, the other is a highly conserved co-receptor, known as Orco^6,7^, in charge of the complex's localization and ion channel formation^8,9^. In some cases, OSN activation requires another set of proteins known as sensory neuron membrane proteins (SNMPs). These proteins are expressed in neurons tuned to lipid-derived pheromone ligands and surrounding cells^10,11^. Detection of non-volatile chemicals (tastants) by sensory neurons of gustatory sensilla is mediated by specialized proteins known as gustatory receptors (GRs). These receptors are also seven-transmembrane domain proteins and, together with the ORs, form the large chemoreceptor superfamily^12^. GRs are notably expressed in antennae, mouthparts, and tarsae, and although they are known to interact mainly with sugars and bitter tastants, some GRs can also detect CO_2_^13^. Finally, there is a second chemosensory receptor family known as the ionotropic receptor (IR) family, whose members belong to the superfamily of ionotropic glutamate receptors, and that are three transmembrane-domain proteins. IRs are involved in both olfaction and taste, detecting acids and amines, but some also intervene in temperature and humidity detection^16,17^. Among IRs, IR8a, IR25a and IR76b are broadly expressed co-receptors that interact with other IRs and form heteromers that respond to an array of specific stimuli^14–17^.

Since discovering the genes encoding ORs in *Drosophila melanogaster*^14–16^, there has been an ever-increasing number of insect species from which genomic and transcriptomic analyses have permitted the description of the molecular machinery used for olfaction and gustation^17^. Considering the relevance of insects for agriculture, many species studied are pests threatening crops worldwide, mainly from Lepidoptera and Coleoptera^18–26^. Despite their economic impact, true weevils (family Curculionidae, subfamily Dryophthorinae) have received less attention, with a few exceptions^27–29^. Among those weevils, *Rhynchophorus ferrugineus* (Oliver), the red palm weevil (RPW), is one of the main threats to date of coconut and oil palms around the world^28^. This species is particularly interesting since its monitoring and control are mainly based on synthetic formulations of male-produced pheromones, constituted by 4-methyl-5-nonanol (ferrugineol) and 4-methyl-5-nonanone (ferrugineone)^30,31^.

Interestingly enough, part of the knowledge in semiochemicals used to manage RPW has been derived from experiences with its American counterpart, *Rhynchophorus palmarum* Linnaeus, also known as the American Palm Weevil (APW)^32,33^. The APW is a devastating pest in oil palm and coconut in the Americas, not only because of the larval feeding (similar to RPW) and vectoring the nematode *Bursaphelenchus cocophilus*, the causal agent of the red ring disease^34–36^. During the 1990s, oil palm production in Latin America was severely affected by this pest until the male-produced pheromone, (*E*)-6-Methyl-2-hepten-4-ol (rhynchophorol), was discovered and utilized as a management tool^32,35,36^. A second step forward for the management of both the RPW and the APW came with demonstrating a synergism between the pheromone blend of each species and the kairomone ethyl acetate the existence of several other kairomones that signal the suitability of the host. This further accentuated the importance of the sense of smell for aggregation and host selection for these species^32,37–39^. Both *Rhynchophorus* species are then almost textbook examples of the use of semiochemicals for pest control, but at the same time continue to represent research avenues due to the menace they pose. The main reason that both members of this genus are so challenging to control rests upon their biological cycle since these insects are confined in the inner parts of their plant hosts, protecting them from any biological and chemical control method^40^. Even though insecticide-based management is reported to be useful to some extent, recent intensive and repeated use of certain insecticides has led to resistance^41^. Thus, there is a need to develop different innovative ways to understand their ecology further and search for promising new technologies for their control. In an attempt to provide information about the molecular actors of the detection of semiochemicals and their putative use for pest control, a recent study identified the most important chemosensory genes involved in olfaction of *R. ferrugineus*^28^. Further research uncovered an OBP and an OR tuned to the palm weevil pheromone^42,43^, opening unprecedented routes for developing OR-OBP-based biosensor arrays and novel mating disruption methods. In the case of *R. palmarum*, the only proteins identified so far are RpalOBP2 and RpalOBP4^44^.

In the present study, we performed a transcriptomic analysis of the antennal tissue of *R. palmarum* to report the main chemosensory-related proteins, i.e., OBPs, CSPs, SNMPs, and the chemoreceptor families, GRs, ORs and IRs. This approach aims to better understand the molecular basis of chemoreception in this pest and eventually develop better control strategies.

## Methods

### Insect collection and antennal tissue dissection

The APW Male and female adults were field-collected in pheromone-baited traps in a commercial oil palm plantation farm located in Coto, South Eastern Costa Rica. All captured insects were transferred to clean plastic containers and fed with sugarcane pieces. Once in the laboratory, the insects were sexed and placed in the freezer (− 8 °C) for about 5 min for immobilization. Directly after, each insect was individually observed under a light microscope, and each pair of antennae was delicately excised with a clean forceps. Antennae from 30 males and 25 females were stored in Eppendorf tubes containing RNA*later* (Themo-Fisher Scientific, WA, USA).

### Total RNA extraction, cDNA library construction, and sequencing

Total RNA was extracted from 12 pairs of antennae from each male and female *R. palmarum*. Excess RNA*later* was removed using sterile filter paper and proceeded to extraction using PureLink RNA Mini Kit (Invitrogen, USA), following manufacturer's instructions. Tissue lysis and homogenization steps were performed at a very low temperature maintained by liquid nitrogen. The quantity and quality of the total RNA were validated using a Qubit 2.0 Fluorometer (Invitrogen, Life Technologies), and the RNA integrity was further confirmed using a 2100 Bioanalyzer (Agilent Technologies). After confirming the quality and the characteristic 'hidden break' in 28S RNA profile using 2100 Bioanalyzer, we proceeded to paired-end cDNA library preparation using TruSeq Stranded mRNA preparation Kit (Illumina Inc.) following manufacturer's protocols, which include the following steps: purification and fragmentation of total RNA, first and second-strand cDNA synthesis, 3′ end adenylation, adapter ligation and purification. Finally, the purified and PCR-enriched products were used for cDNA library preparation. The cDNA libraries were validated and quantified by Qubit 2.0 Fluorometer. The HiSeq Illumina sequencing was performed at the core sequencing facility of the King Abdullah University of Science and Technology (KAUST), Jeddah, Saudi Arabia. Image deconvolution and quality value calculations were performed using Illumina GAPipeline1.3.

### Data processing, assembly, and gene annotation

Data processing, transcriptome assembly, and functional annotation were carried out by following the method described previously^28^, and contigs were identified and annotated based on local Blast search using *R. ferrugineus* chemosensory protein sequences, using Geneious *v*7.1.5 (http://www.geneious.com). The top blast hit transcript clusters were extracted from the male and female assembled transcriptomes with an in-house command-line script. Relevant sequences were translated and manually selected based on the following criteria: e-value score below 1.0E−5, similarity to other chemosensory proteins, and with an ORF with at least 50% the average length traditionally observed for each gene family. Selected sequences were numbered according to their estimated expression, which was obtained by RPKM values^45^. To assess the transcriptomes' completeness, an Arthropoda BUSCO database, consisting of 1066 core genes that are highly conserved single-copy orthologues^46,47^, was used to query the transcriptome fasta files. For this process, the gVolante web server (https://gvolante.riken.jp/) was utilized with the following parameters: min_length_of_seq_stats: 1, assembly_type: trans, Program: BUSCO_v2/v3, selected reference_gene_set: Arthropoda.

### Phylogenetic analysis of the candidate chemosensory proteins

To predict phylogenetic relationships between the selected sequences, available relevant chemoreceptor protein sequences were retrieved from GenBank. For OBPs, predicted sequences were compared with those from *Anomala corpulenta*, *A. cuprea*, *A. octiescostata*, *Colaphellus bowringi*, *Cyrtotrachelus buqueti*, *Galeruca daurica, Holotrichia oblita*, *Popilio japonica*, *R. ferrugineus*, *Tenebrio molitor*, *Tomicus yunnanensis*, *Tribolium castaneum*, and DmelLUSH as outgroup. For CSPs, the novel sequences were compared with those from *Agrilus planipennis*, *Anoplophora glabripennis*, *Dendroctonus ponderosae*, *R. ferrugineus*, and *Bombyx mori*. In the case of SNMPs, putative sequences of *R. palmarum* were compared with those from *A. glabripennis, B*. *mori, Cylas formicarius*, *D. ponderosae* and *R. ferrugineus.* For GRs, the sequences predicted from *R. palmarum* were compared with the sets of putative GRs from *C. formicarius D. ponderosae* and *R. ferrugineus.* For ORs, predicted *R. palmarum* sequences were compared with those from *C. formicarius, D. ponderosae, I. typographus, M. caryae* and *R. ferrugineus* using *M. caryae* co-receptor as outgroup. Finally, for IRs, sequences from *A. glabripennis, D. melanogaster, D. ponderosae* and *R. ferrugineus* were used to compare with those predicted from *R. palmarum*. In all cases, predicted sequences were aligned using MAFFT web-based version 7.220^48^, with default parameters. Aligned sequences were used to calculate the best substitution model for each gene family in ProtTest 3.4^49^. The selected model was used to construct maximum likelihood trees using RAxMLGui 2.0^50^, with branch support calculated by rapid bootstrap (N = 100). The trees were visualized and edited with iTOL^51^, FigTree *v*1.4 (tree.bio.ed.ac.uk), and colored and finally edited with Adobe Illustrator (Adobe, CA, USA).

## Results

### General results of the de novo assembly

De novo transcriptomes were assembled for each male and female antennae of the American palm weevil adults. The raw reads were deposited at the National Center for Biotechnology Information (NCBI) Sequence Read Archive (SRA) database with the accession SRR12450122 and SRR12450123, respectively for the male and female *R. palmarum*. The transcriptome Shotgun Assembly (TSA) project was deposited at DDBJ/EMBL/GenBank under the accession GIUZ00000000 (BioProject: PRJNA656150; BioSample: SAMN15768540). In the case of the male transcriptome, a total number of 398,485,158 raw reads were generated. The total number of clean reads was 237,598,785, which yielded a total of 56,786 contigs, with an average length of 679 bp, an N50 length of 1098 bp, and a GC content of 36.84% (Table 1). In the transcriptome assembled from the female antennae, a total number of 304,666,926 raw reads were generated. The total number of clean reads was 186,448,624, which yielded a total of 53,791 contigs, with an average length of 689 bp, an N50 length of 1162 bp, and a GC content of 36.95%. BUSCO analysis was performed separately on each male and female transcriptomes. It resulted in hits for 97.19% of queried sequences for both transcriptomes, 88.9%, and 88.8% identified as complete in male and female transcriptomes, respectively, indicating satisfactory completion of the two transcriptomes. A comparison between the transcriptome assembled for *R. ferrugineus*^28^ and the average numbers obtained for both sexes of *R. palmarum* is shown in Table 1.

**Table 1:** Comparative summary of antennal transcriptome assemblies of two palm weevil species.

	*R. palmarum* Male	*R. palmarum* Female	*R. ferrugineus*^32^
Total number of raw reads	398,485,158	304,666,926	194,157,678
Total length of reads (bp)	60,171,258,858	46,004,705,826	
Total number of reads cleaned	237,598,785 (222,750,886 in pairs)	186,448,624 (176,219,320 in pairs)	183,355,534
Total length of reads cleaned (bp)	34,092,680,247	26,985,654,111	
Number of contigs	56,786	53,791	35,667

### GO analysis and transcript abundance

Male and female antennal transcriptomes of *R. palmarum* were used as BLASTx queries against the non-redundant NCBI protein database and were subjected to InterProScan analyses. For most transcripts of both male and female antennal transcriptomes, the greatest number of significant blast hits were to sequences of *D. ponderosae*, followed by *Sitophilus oryzae* and *Tribolium castaneum* (Supplementary Figure S1a and S1b). Top-blast hits were substantially distributed to *S. oryzae* (12,881 and 12,730 in male and female, respectively), likely reflecting the degree of relatedness between these two species in the context of genetic information that is available in the NCBI database. BLASTx and InterProScan results were utilized for functional GO annotations. For both transcriptomes, a majority of GO annotations were derived from Interpro and Uniprot databases (Figure S1a and S1b).

For the male transcriptome, Blast2Go (B2G) and InterProScan analyses were performed on 56,786 transcripts. Blast hits were identified for 24,106 transcripts, of which 4041 yielded no GO annotation, 4504 were assigned GO terms but could not be functionally annotated, and 15,561 were B2G annotated. (Figure S1a). For the female transcriptome, B2G and InterProScan analyses were performed on 53,791 transcripts. Blast hits were identified for 23,455 transcripts, of which 3873 yielded no GO annotation, 4115 were assigned GO terms but could not be functionally annotated, and 15,467 were B2G annotated (Figure S1b).

For both male and female transcriptomes, level 2 GO term distributions were consistently ranked. Within the “biological processes” GO ontology, the “cellular process”, “metabolic process”, and “biological regulation” terms were the most abundantly assigned to the transcripts. In the “molecular function category”, “binding” and “catalytic activity” were the most abundant assignments. In the “cellular components” category, “cellular, the anatomical entity” and “intracellular” were the most abundant assignments (Figure S1a and S1b).

RPKM based transcript abundance calculation in each male and female transcriptome revealed several uncharacterized proteins as most abundant transcripts in both, followed by heat shock 68-like proteins (Supplementary Table S1). Within the chemosensory gene families, OBPs were the most abundant group, with RpalOBP2 being the most abundant transcript in both males (RPKM: 5658.70) and female (RPKM: 11,523.09) transcriptome.

### Odorant-binding proteins (OBPs)

**Figure 1:**
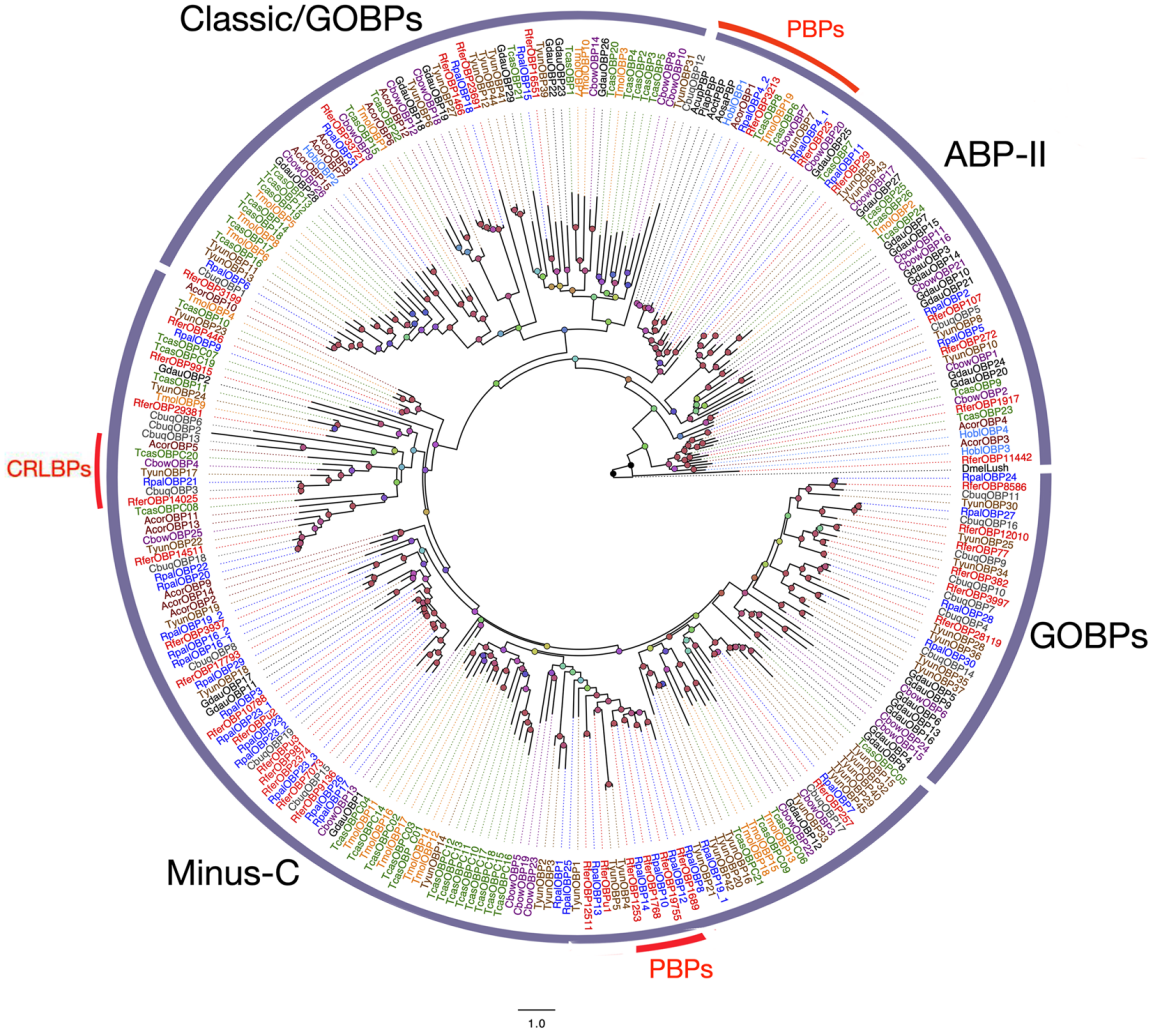
Maximum likelihood tree of the odorant-binding proteins (OBPs) predicted from the antennal transcriptome of *R. palmarum* and from several other species. Unrooted. Includes sequences from *Anomala corpulenta* (Acor), *A. cuprea* (Acup), *A. octiescostata* (Aoct), *Colaphellus bowringi* (Cbow), *Cyrtotrachelus buqueti* (Cbuq), *Galeruca daurica* (Gdau)*, Holotrichia oblita* (Hobl), *Popilio japonica* (Pjap), *R. ferrugineus* (Rfer), *Tenebrio molitor* (Tmol), *Tomicus yunnanensis* (Tyun), *Tribolium castaneum* (Tcas), and *Drosopila melanogaster* (DmelLUSH). The best substitution model calculated and used corresponded to LG + G4. Node colours represent bootstrap support (n = 100) with gradient green to red representing bs 40 to 100. OBP subfamilies Minus-C, ABPII, CRLBP (Red), and classic/GOBPs/PBPs are labeled. Sequence names of *R. ferrugineus* and *R. palmarum* have been colored in red and blue font, respectively. Two clusters with well-characterized pheromone-binding OBPs (PBPs) and one CRLBP clade are marked in red.

We identified 37 candidate OBPs in the antennae of APW adults through our qualitative transcriptome analysis, 22 of which presented full-length open reading frames (ORFs). The average sequence length of the annotated OBPs was 853 bp. Four candidates OBPs (RpalOBP8, 9, 12, and 16.1) were not found in the female transcriptome, whereas only one (RpalOBP16.2) was absent from the male transcriptome. Except for OBP2 and paralogs (4.1 and 4.2), all other OBPs were named accordingly to the relative transcript abundance, as reported in the case of *R. ferrugineus*^42^, in which OBP2 and OBP1 were the most highly abundant transcripts followed by OBP3 and OBP4 paralogs. In contrast, all the other predicted OBPs were at least four times less abundant than these four (Table S2). The phylogenetic analysis confirmed the closeness between RpalOBP2, RpalOBP4.1, and RpalOBP4.2 and their orthologs from *R. ferrugineus*, RferOBP107, RferOBP23, and RpalOBP3213, respectively, with all of them clustering together in the so-called “antennal binding proteins II (ABPII)” subfamily of OBPs (Fig. 1). The RpalOBP4.1 *R. ferrugineus* ortholog, RferOBP23, is a highly expressed OBP previously classified as a candidate PBP as its silencing slightly impaired ferrugineol detection^42^. Both RpalOBP4.1 and RferOBP23 belong to a phylogenetic cluster that includes other well studied pheromone-binding proteins like PjapPBP^52^. RpalOBP1, being the most highly expressed OBP, clustered with a previously characterized antennal-specific RPW PBP, RferOBP1768^42^. They defined another phylogenetic OBP cluster within the Minus-C family, although they shared less than 25% sequence identity. Furthermore, several other OBPs from *R. palmarum* clustered together in this group along with other RferOBPs. Within this cluster, RpalOBP10 shared close sequence similarity (84% identical residues) with the PBP RferOBP1768. RpalOBP21 was the only RpalOBP that grouped in the CRLBP subfamily, sharing 87.02% identity with RferOBP14025. We observed that around two thirds of the predicted RpalOBPs belonged to Minus-C subfamily with the characteristic absence of C2 and C5 cysteine residues (Figure S2), five were ABP-II subfamily members (RpalOBP2, 5, 4.1, 4.2, 11) four were classic OBPs (RferOBP6, 15, 31, 18) and remaining were classified as GOBPs/PBPs (Fig. 1).

### Chemosensory proteins (CSPs)

**Figure 2:**
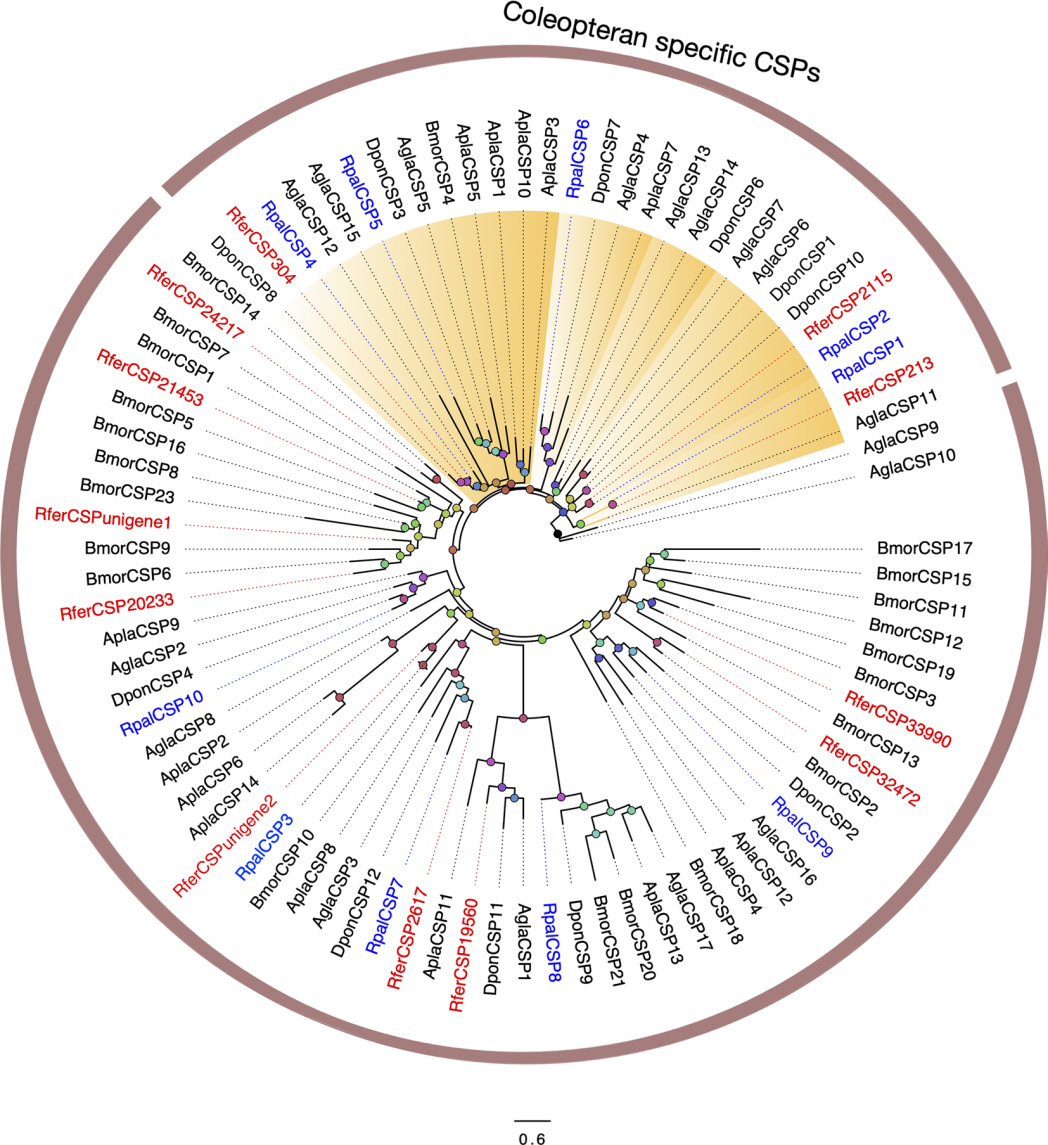
Maximum likelihood tree of the chemosensory proteins (CSPs) predicted from the antennal transcriptome of *R. palmarum* and from several other species. Unrooted. Includes sequences from *Agrilus planipennis* (Apla), *Anoplophora glabripennis* (Agla), *Dendroctonus ponderosae* (Dpon), *R. ferrugineus* (Rfer), and *Bombyx mori* (Bmor). The best substitution model calculated and used corresponded to LG + G + I. Numbers at nodes represent bootstrap support (n = 100) and are only shown if ≥ 75. The coleopteran specific cluster is colored in orange. Sequence names of *R. ferrugineus* and *R. palmarum* have been colored in red and blue font, respectively.

A total of 10 CSPs were identified in the antennal tissue of APM adults, eight of which with full-length ORFs. All the detected candidate genes were found in both male and female transcriptomes. For the predicted CSPs, an average sequence length of 950 bp was obtained. This number of detected RpalCSPs is similar to what has been observed in its closest relative, *R. ferrugineus* (12 CSPs), or in other antennal transcriptomes in more divergent Coleoptera, such as *Tenebrio molitor* (12), *Rhyzoperta dominica* (8), and *D. ponderosae* (6)^22,23,28,53^. Except for RpalCSP7, all of these CSPs contained the conserved pattern of four cysteine residues (Cys-X6-Cys-X18-Cys-X 2-Cys, where X represents any amino acid) (Figure S3). The most highly expressed contig (Table S3), RpalCSP1 (with 2239.41 RPKM), shares 91.5% identity with the RferCSP213 from *R. ferrugineus*, whereas the second most abundant, RpalCSP2, shares 92.0% with RferCSP2115. These sequences, in addition to RpalCSP4, RpalCSP5 and RpalCSP6, clustered together with several coleopterans specific CSPs, a feature also observed with some RPW CSPs. Most RpalCSPs are related to sequences from *D. ponderosae* or *R. ferrugineus* (Fig. 2)*.* Interestingly, RpalCSP4 shares a 93.8% identity with its correspondent ortholog in RPW, RferCSP304. Other closely related sequences identified in both transcriptomes are RpalCSP3 with RferCSPunigene2 and RpalCSP7 with RferCSP2617 that share 76.7 and 75.3%, respectively.

Regarding the different CSPs found within *R. palmarum* antennae, the percentage of identical residues ranged from 17.4 to 62%, indicating how divergent this gene family is. However, as it is characteristic of this gene family, predicted APW CSPs were very well conserved between species, with at least five out of ten CSPs corresponding to closely related RPW orthologs with percentages of identical residues 75–94%. Still, several of the other RpalCSPs clustered with CSPs from *D. ponderosae* and *A. plannipenis* rather than with those from RPW (Fig. 2).

### Sensory neuron membrane proteins (SNMPs)

**Figure 3:**
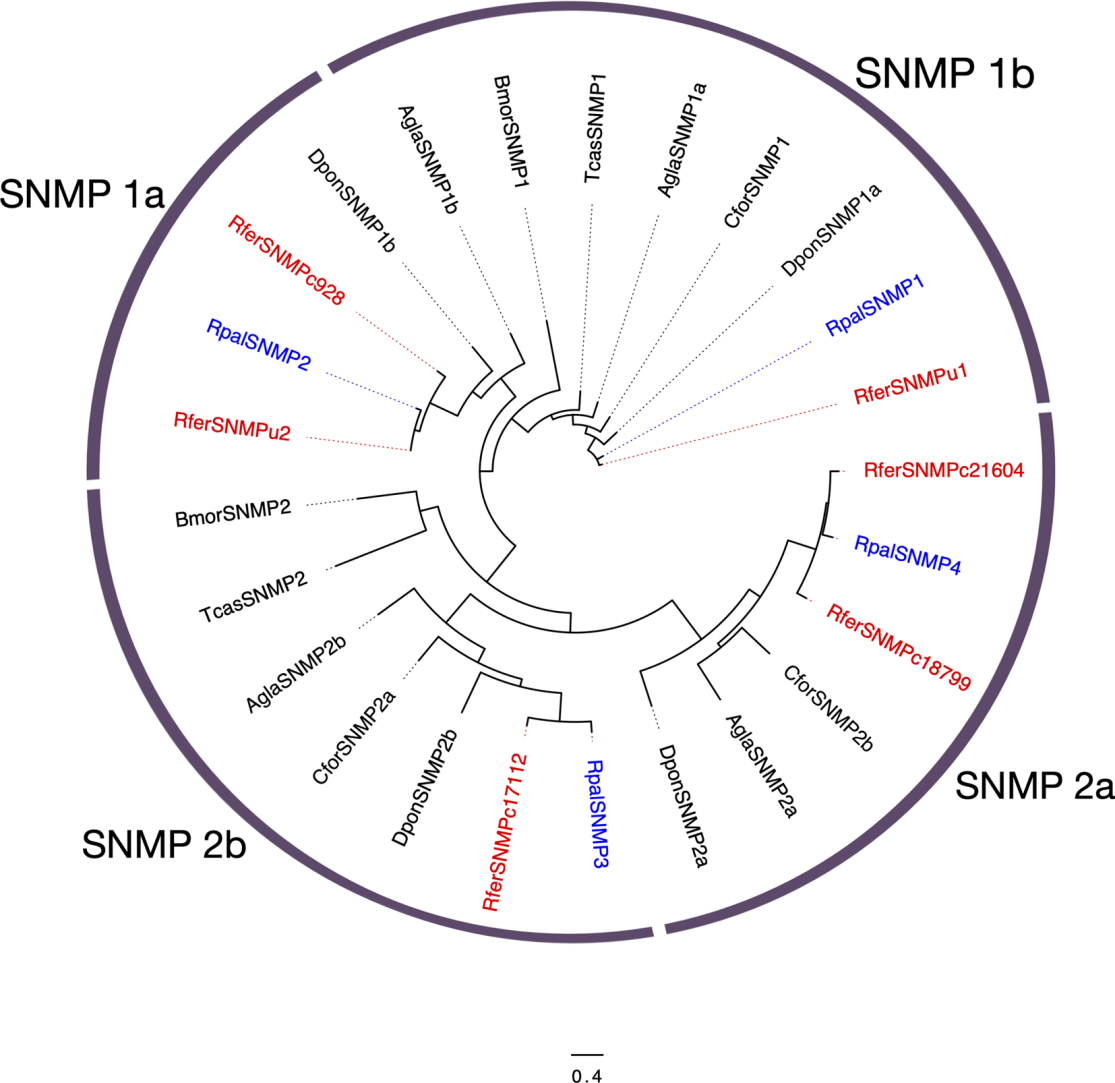
Maximum likelihood tree of the sensory neuron membrane proteins (SNMPs) predicted from the antennal transcriptome of *R. palmarum* and from several species. Unrooted. Includes sequences from *Anoplophora glabripennis* (Agla)*, B*. *mori* (Bmor)*, Cylas formicarius* (Cfor), *Dendroctonus ponderosae* (Dpon) and *R. ferrugineus* (Rfer)*.* The best substitution model calculated and used corresponded to LG + G. Numbers at nodes represent bootstrap support (n = 100) and are only shown if ≥ 75. SNMP subfamilies are marked. Sequence names of *R. ferrugineus* and *R. palmarum* have been colored in red and blue font, respectively.

The group of proteins known as SNMPs belongs to the CD36 superfamily of proteins in insects, which are functionally associated to signal facilitation in response to external stimuli, in the case of SNMPs meaning environmental chemical stimuli^54^. Our analysis determined four candidate SNMPs in *R. palmarum* adults' antennae in both male and female transcriptomes, with only one candidate showing a full-length ORF. The sequences found presented an average length of 2594 bp. These sequences were highly divergent among them (15.3–28.3% identical residues). High percentages of identical residues and strong bootstrap support imply the proximity between the orthologs of both APW and RPW species (Fig. 3). The most abundant contig (Table S4), RpalSNMP1 (RPKM: 882.57), the shared identity of 88.3% with its putative ortholog in *R. ferrugineus,* RferSNMPu1. Both APW and RPW SNMP1 sequences clustered in the subfamily of SNMPs known as SNMP1 proteins, which is divided into SNMP1a and SNMP1b. Whereas RpalSNMP1 belongs to the “a” division, RpalSNMP2 likely belongs to the “b” division of the SNMP1 subfamily. The second most abundant transcript, RpalSNMP2, shared 75.6% identity with RferSNMP928. RpalSNMP3 and 4 showed 79 and 77% identity with their closest putative orthologs, RferSNMP17112 and RferSNMP18799, respectively. According to our phylogenetic analysis, RpalSNMP3 and RpalSNMP4 can be classified as part of the SNMP2 subfamily.

### Gustatory receptors (GRs)

**Figure 4:**
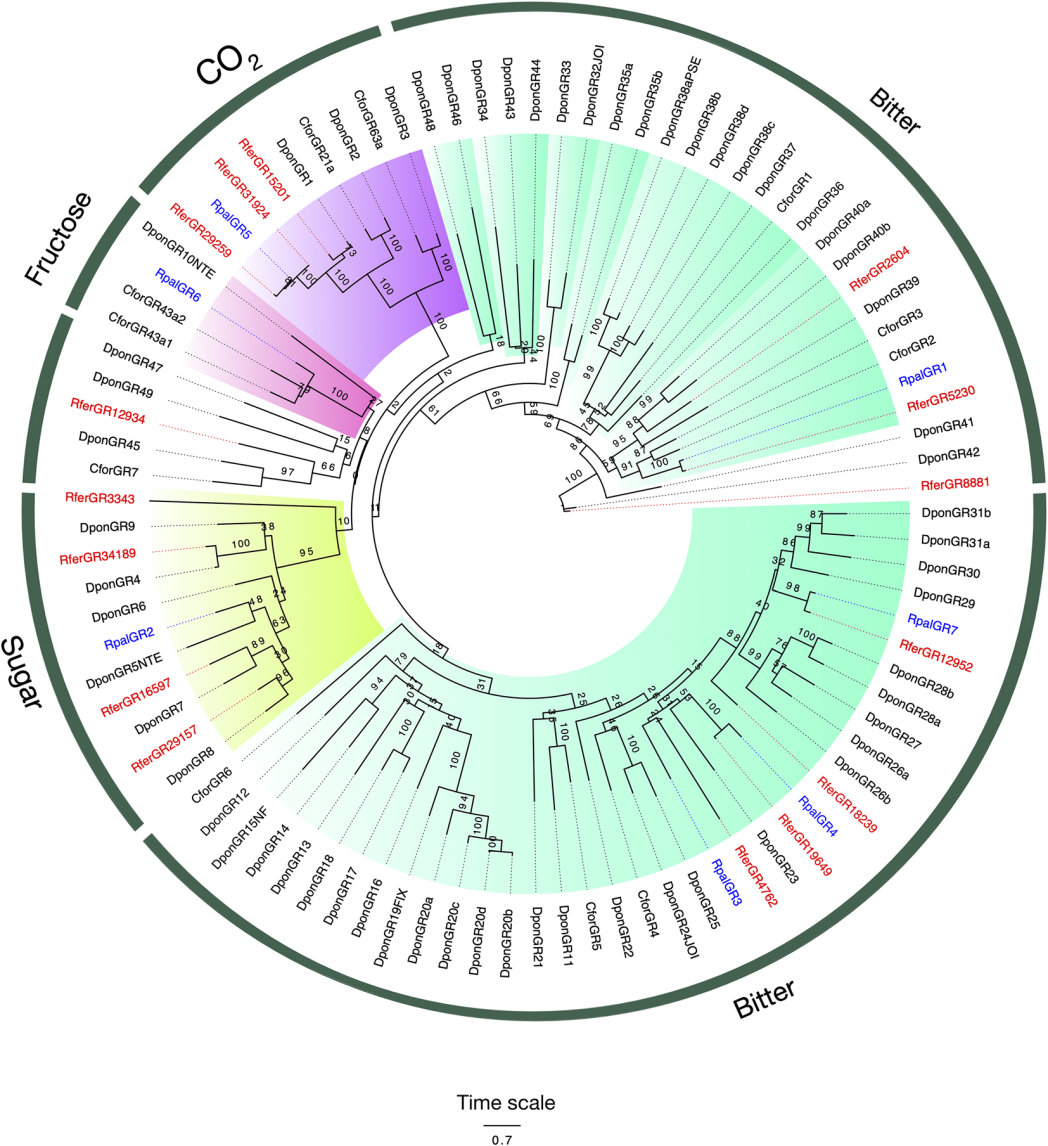
Maximum likelihood tree of the gustatory receptor proteins (GRs) predicted from the antennal transcriptome of *R. palmarum* and from several species. Unrooted. Includes sequences from *C. formicarius* (Cfor)*, D. ponderosae* (Dpon) and *R. ferrugineus* (Rfer). The best substitution model calculated and used corresponded to JTT + G + F. Numbers at nodes represent bootstrap support (n = 100) and are only shown if ≥ 75. GR subfamilies are named and colour coded as follows: purple for candidate CO_2_ tuned receptors, pink for putative fructose receptors, green for putative conserved general sugar receptors, and dark green for putative bitter receptors. Sequence names of *R. ferrugineus* and *R. palmarum* have been colored in red and blue font, respectively.

Whereas 15 GRs have been previously identified in the RPW antennal transcriptome, only seven-candidate GRs were found expressed in the antennae of APW adults, with only three of them showing full-length ORFs. The average length was 1537 bp. Interestingly, one single candidate GR (RpalGR2) was found only in the female transcriptome, whereas the rest were present in both sexes. The conserved C-terminal motif of “TYhhhhhQF”, characteristic of GRs, was found in five of the seven predicted GRs (Figure S4). The most abundant transcript (Table S5) from this family of receptors (RpalGR1, with 25.98 RPKM) clustered together with receptors tuned presumably to bitter compounds. Other predicted receptors such as RpalGR3, RpalGR4, and RpalGR7 were grouped in the same clade. RpalGR2 clustered within a group of conserved candidate sugar receptors, while RpalGR6 clustered with what has been mentioned as a fructose receptor in *D. ponderosae.* RpalGR5 clustered in a very conserved and strongly bootstrap-supported group of receptors putatively tuned to CO_2_ (Fig. 4). Interestingly, RpalGR2 and RpalGR6 were the only receptors for which direct orthologs were not identified in the antennal transcriptome of *R. ferrugineus*, whereas RpalGR1, RpalGR3, RpalGR4, RpalGR5, and RpalGR7 presented identity percentages with RferGRs that range between 23.9 and 95.8%. Despite the fact that the low number of GRs identified, the phylogeny showed that the RpalGRs are well distributed among the different functional clades, with at least one GR found in each type (Fig. 4).

### Odorant receptors (ORs)

A total of 63 novel putative odorant receptor-encoding transcripts were annotated from the APW adult antennal transcriptome, with 32 of the predicted ORs showing full-length ORFs. The average sequence length of the annotated ORs was 1423.7 bp. Only one predicted OR was found exclusively in the male transcriptome (RpalOR64), and similarly, only one (RpalOR55) was solely found in the female transcriptome. Our results show that from 63 RpalORs identified, at least 57 had a corresponding putative ortholog from *R. ferrugineus.* As expected, the most abundant contig (with 124.10 RPKM, Table S6) corresponded to the most conserved one: the olfactory co-receptor protein (Orco). Here again, both palm weevils' genetic proximity is explicitly demonstrated since RpalOrco and RferOrco share 98.7% of identical residues. In contrast, identity with the rest of the corresponding orthologs from other species (*D. ponderosae, I. typographus*, *C. formicarius, M. caryae*) ranges from 87.7 to 89.6%.

**Figure 5:**
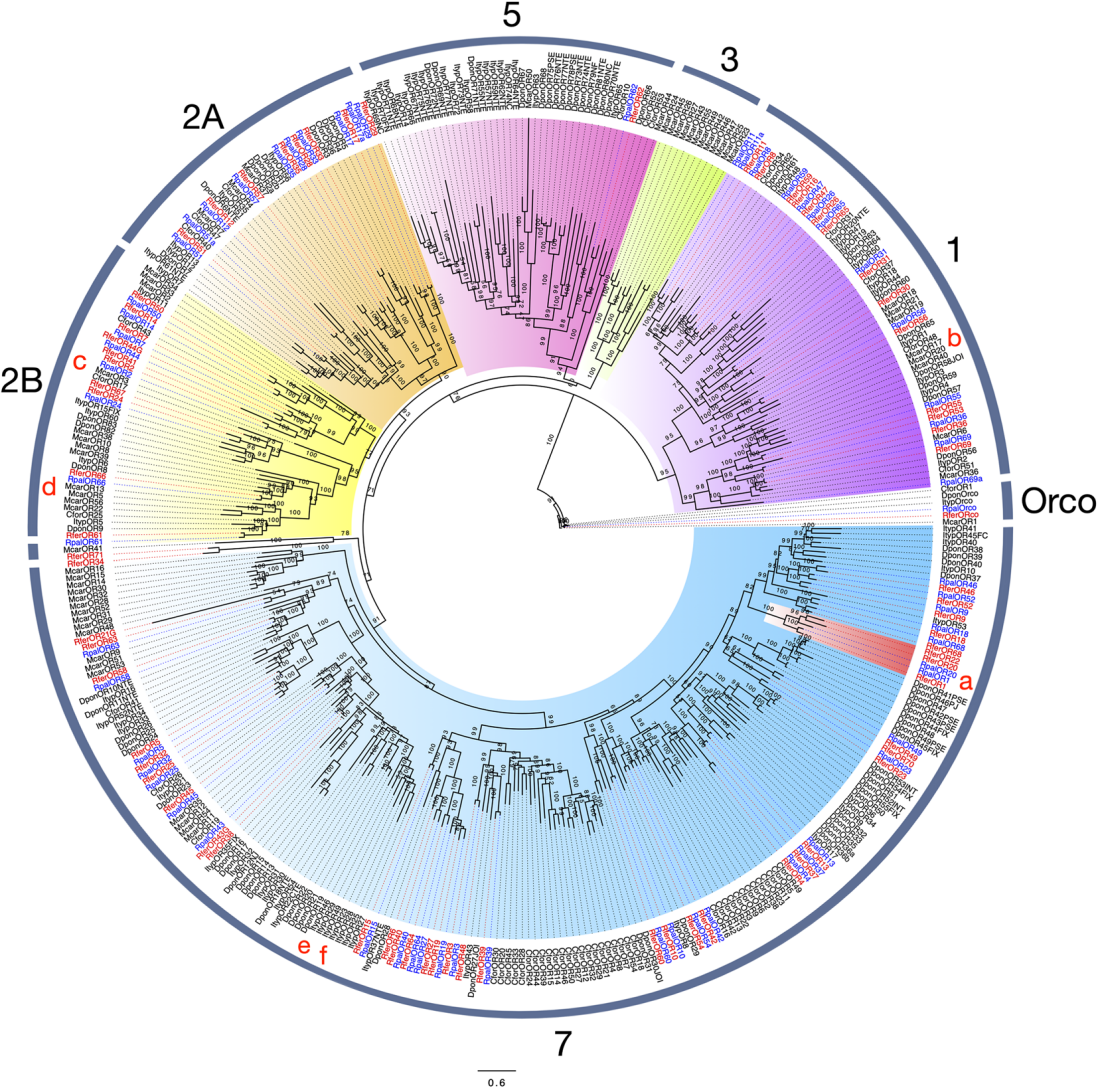
The maximum likelihood tree of the odorant receptor proteins (ORs) is predicted from the antennal transcriptome of *R. palmarum* and from several species. Includes sequences from *C. formicarius* (Cfor)*, D. ponderosae* (Dpon)*, I. typographus* (Ityp*, M. caryae* (Mcar) and *R. ferrugineus* (Rfer). The odorant coreceptor McarOR1 is used as outgroup. The best substitution model calculated and used corresponded to JTT + G + F. Numbers at nodes represent bootstrap support (n = 100). OR subfamilies are numbered, and clades are colour coded as follows: purple for subfamily I, orange for subfamilly 2A, yellow for subfamily 2B, green for subfamily III, pink for subfamily V, light blue for subfamily VII. A pheromone receptor clade identified: RferOR1 (**a**) from *R. ferrugineus*^43^; three ORs McarOR20 (**b**), McarOR3 (**c**) and McarOR5 (**d**) from *M. caryae*^26^; and two ORs ItypOR46 (**e**) and ItypOR49 (**f**) from *I. typographus*^80^. Sequence names of *R. ferrugineus* and *R. palmarum* have been colored in red and blue font, respectively.

Considering the nine monophyletic families suggested by Mitchell et al.^55^, and the previous classification^28^, the predicted RpalORs distributed among all the families except for families III, IV, and VI (Fig. 5), as RferORs did. Within the 63 APW ORs, including Orco, most of the predicted receptors clustered in four subfamilies: 13 ORs in subfamily I, 18 ORs in subfamily II, one (RpalOR62) in subfamily V and the remaining 30 ORs in subfamily VII.

When looking at proposed pheromone receptors (PRs) from *R. ferrugineus*^28^*,* it becomes clear that APW shared some closely related orthologs: the sequence RferOR43 and RpalOR43 shared 85% identity, RferOR44 and RpalOR44 shared 78% identity, and RferOR63 and RpalOR62 shared 69% identity. However, none of these proposed RPW pheromones ORs have yet been functionally characterized, but a recent report identified RferOR1^43^ as the aggregation pheromone receptor in the RPW. Our phylogeny identified a potential functional ortholog in APW, RpalOR1 that shared 82.24% amino acid identity with RferOR1. RpalOR20 was also found in the same phylogenetic cluster as RpalOR1 and RferOR1, potentially defining a palm weevil pheromone receptor clade (Fig. 5).

### Ionotropic receptors (IRs)

**Figure 6:**
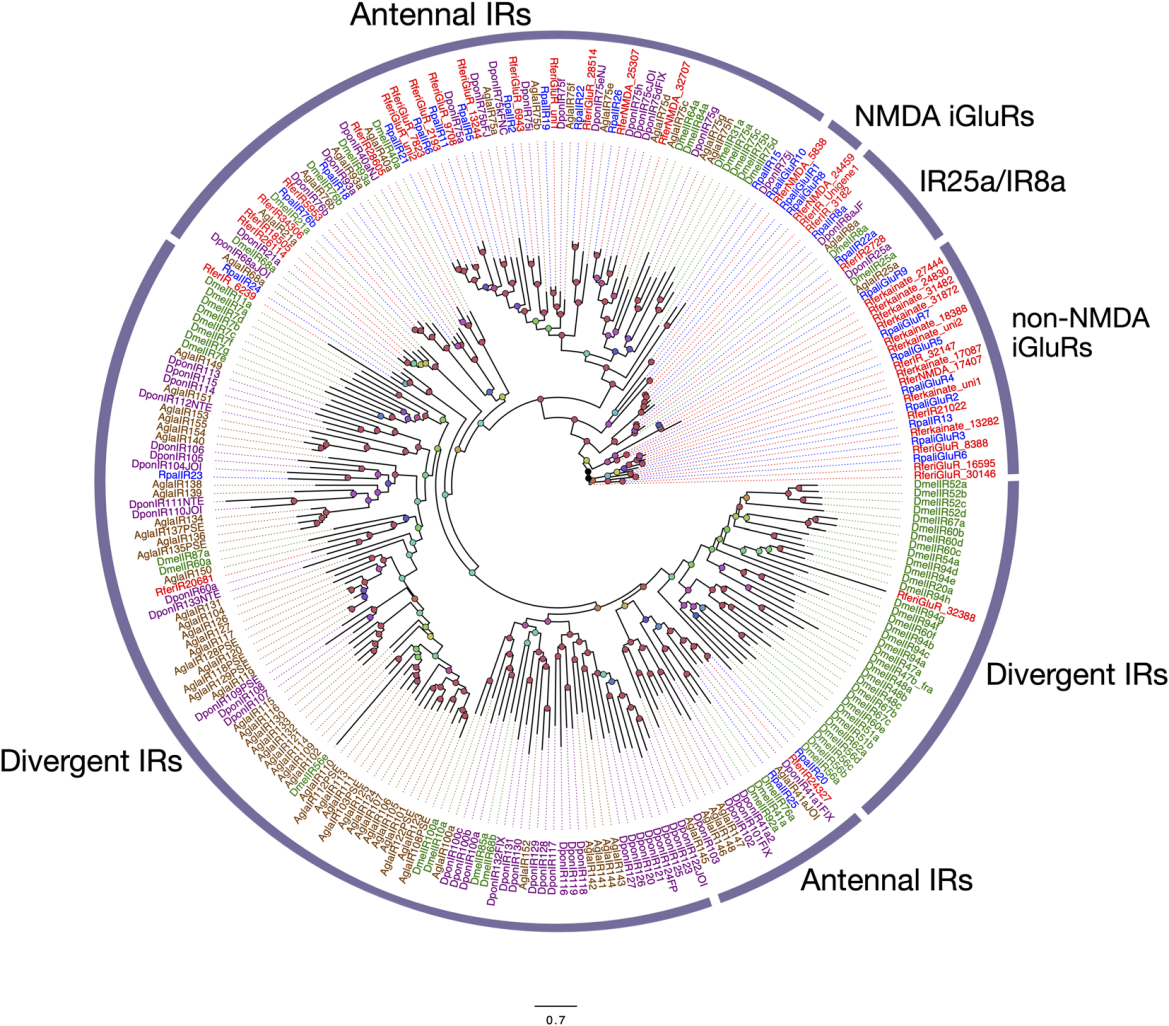
The maximum likelihood tree of the ionotropic receptor proteins (IRs) is predicted from the antennal transcriptome of *R. palmarum* and from several species. Unrooted. Includes sequences from *Anoplophora glabripennis* (Agla)*, Drosophila melanogaster* (Dmel)*, D. ponderosae* (Dpon) and *R. ferrugineus* (Rfer). The best substitution model calculated and used corresponded to WAG + G + F. Numbers at nodes represent bootstrap support (n = 100) and are only shown if ≥ 75. Sequence names of *R. ferrugineus* and *R. palmarum* have been colored in red and green font, respectively.

We identified a total of 28 putative IRs from the antennal transcriptome of *R. palmarum,* 10 of them with full-length ORFs*.* The average sequence length of the putative receptors was 2412.5 bp. The most highly abundant contig was named RpalIR8a with 32.82 RPKM (Table S7) since it was closer to DponIR8a and DmelIR8a than to other candidates IRs. Similarly, RpalIR25a was named after its percentage of identical residues with DmelIR25a (78.75%) and DponIR25a (92.52%), and RpalIR76b was named after its putative ortholog DponIR76 (69.3% identical residues). These three receptors are putatively broadly expressed co-receptors. At least ten predicted sequences were categorized as ionotropic glutamate receptors (iGluRs), three of them grouped with RPW candidates to be N-methyl-D-aspartame (NMDA) receptors, whereas the rest were classified as non-NMDA iGluRs. One single predicted receptor was classified in the group of divergent IRs, whereas the rest were classified as antennal IRs (Fig. 6).

## Discussion

Palm weevils *R. ferrugineus* and *R. palmarum* are invasive pests responsible for millions of economic losses. One of the current management approaches is based on placing pheromone blends (ferrugineol, rhynchophorol) and kairomones (ethyl acetate, ethanol, acetoin, and sugar ferments) on traps that allow capturing as many insects as possible, a strategy called mass trapping^30,35–40,56–58^. Fortunately, behavioral and ecological similarities between these closely related species have permitted the development of dual management technologies^33^. However, misuse of mass trapping sometimes fails to protect all the palms in a plantation, and oviposition by these insects usually associates with immediate palm loss. In this sense, the possibility to develop new management strategies based on “reverse” chemical ecology by targeting proteins with chemosensory function holds promise as a futuristic and elegant way to disable insect host and mate finding. Furthermore, understanding the molecular machinery of chemoreception can also help understand insect behavioral specializations based on genetic divergence^59,60^.

A total of 37 OBPs, 10 CSPs, 4 SNMPs, 7 GRs, 63 ORs, and 28 IRs have identified in our APW antennal transcriptomes. Except for two OBPs, all the sequences identified are entirely novel. Compared to the antennal transcriptome analysis provided for *R. ferrugineus*, our transcriptome analysis for *R. palmarum* presented a larger number of reads and contigs identified (Table 1). Interestingly, only five OBPs, one GR, and two ORs were differentially expressed between sexes. Since this species utilizes an aggregation pheromone blend detected by both males and females, it is expected that both sexes share common grounds for most of their life cycles. However, the existence of differentially expressed chemosensory transcripts may indicate the detection of important cues for sex-specific activities such as oviposition. However, due to the lack of the functional characterization of any of these transcripts, the meaning of such difference remains unknown.

In the APW antennal transcriptome, the most abundant chemosensory transcripts were determined as putative OBPs, particularly RpalOBP2 and RpalOBP1. Within RpalOBPs, we pinpointed putative APW PBPs, based on their high expression (RpalOBP2) and/or phylogenetic position close to characterized coleopteran PBPs (RpalOBP4.1, 4.2, 10, 12, and 14). The most promising candidate consisted of RpalOBP10, as it is the closest ortholog of a functionally confirmed RPW PBP, RferOBP1768^42^, and both share 84.07% identical residues. Both OBPs are members of the Minus-C subfamily, which has been proposed in the past to contain pseudogenes, non-olfactory OBPs, or a novel type of OBPs characteristic from Coleoptera^61^. The functional characterization of RferOBP1768 demonstrates that at least its corresponding gene is not a pseudogene. Another interesting APW PBP candidate consists of RpalOBP6, as it is closely related to one OBP of the bamboo snout beetle *Cyrtrotrachelus buqueti*, which has been shown to strongly binds to the pheromone analog dibutyl phthalate, and much less to general plant volatiles^62^. Further functional characterization work with these candidates is needed, considering that OBPs and ORs are candidates to be used as targets for management techniques^42,43^.

Looking at CSPs, it is interesting to note that APW lacks some CSPs in RferCSP-defined clades. It is possible that we missed these CSPs in the APW transcriptome, but given this transcriptome coverage compared to the RPW one, a more plausible interpretation is that both species have evolved different sets of CSPs. Hypothesizing these proteins' specific function is daunting and probably impossible, especially considering that CSPs' functional characterization has been demonstrated only through fluorescent binding assays of CSPs from species of other insect orders^63–66^. Furthermore, evidence indicates that these proteins may have different non-olfactory roles in embryonic development and leg regeneration^67,68^.

Our transcriptome analysis found four SNMPs, all of them presenting closest homology to *R. ferrugineus* SNMPs (Fig. 3). Several works on the SNMP subfamily showed that SNMP1 proteins are usually highly expressed in pheromone receptor neurons, indicating their putative involvement in pheromone detection, a function later evidenced in *D. melanogaster* and more recently in *B. mori*^10,69,70^. However, whether the weevil SNMP1s are indeed involved in detecting pheromone components remains to be investigated, especially considering that the function of SNMPs has not been evidenced in any Coleoptera. RpalSNMP3 and RpalSNMP4 clustered in the SNMP2 subfamily, which leads us to hypothesize that they may be expressed in supporting cells surrounding OSNs in antennae, legs, and wings, as it has been observed in other insects^71–73^, and are probably involved in different functions than pheromone detection.

Our transcriptome analysis focused only on antennal expression when it comes to gustatory receptors, which is why we found solely seven GRs. In contrast, genome analyses of other Coleoptera such as the emerald ash borer and the mountain pine beetle identified 30 and 60 GRs, respectively^61^. The number of predicted RpalGRs was lower than that in the RPW antennal transcriptome (15)^28^. The highest percentage of identical residues between RPW and APW GRs was observed between RpalGR5 and RferGR29259, both clustering in the putatively CO_2_ tuned GR clade, a highly conserved clade across insect orders. These receptors' suggestion may be responsive to CO_2_ is relevant according to these palm weevils' ecology. Several works on this genus have shown that fermenting compounds from plant tissue synergize the behavioral response to different Rhynchophorus species' pheromone, probably because these compounds indicate damaged palms as suitable places to find female partners for males and oviposition sites for females^74^. As a blatant product of fermentation, carbon dioxide could be part of the chemical signatures that these insects use to find the host. Sugars may also be important cues for host identification, and our transcriptome identified at least 2-candidate sugar/fructose GRs in APW, although more have been annotated in RPW. However, speculation on the function of these and other GRs is difficult, as no coleopteran GRs have been characterized to date.

Looking at ORs, we identified RpalORs in all the protein subfamilies that contained RferORs (Fig. 5). The RpalOR distribution in these subfamilies was similar to that of RferORs, *D. ponderosae* ORs*,* and *I. typographus* ORs, with an abundance of ORs subfamilies I, II, and VII, which may indicate some OR expansion or specialization in these species. It is important to notice that despite the phylogenetic relatedness between both palm weevils, we evidenced at least four ORs that may represent independent expansions in *R. palmarum* (RpalOR69a and 17a) (Fig. 5). This is not wholly unexpected since, despite the similarities between both species, they are distributed in different geographical locations whose characteristics may act as drivers of genetic differences in chemoreception. More information will come from further OR functional characterization. Indeed, only a small number of Coleoptera OR functional studies have been conducted, identifying mainly pheromone receptors (Fig. 5). Anyhow, this can serve as a basis to propose candidate PRs in the APW. The most promising APW PR candidate is RpalOR1, closely related to the sole *Rhynchophorus* OR characterized to date, namely RferOR1. Further confirmation of the RpalOR1 function in the APW pheromone detection would be an exciting finding since the APW and RPW do not share the same pheromones. APW responds to rhynchophorol (2(*E*)-6-methyl-2-hepten-4-ol), whereas RPW utilizes a blend of ferrugineol (4-methyl-5-nonanol) and ferrugineone (4-methyl-5-nonanone)^30,31,56,75^. RferOR1 has been shown to be tuned towards ferrugineol and ferrugineone, but not to rhynchophorol^43^. If RpalOR1 appears to have opposite tuning, this will open the way to structure–function relationship studies, the ~ 20% amino acid difference altering the pheromone-binding*.* In *R. palmarum*, electrophysiological work has shown the existence of some OSNs whose responses to pheromone are synergized by acetoin stimulation^76^. Further functional work on RpalOR1 would allow a better understanding of some kairomones' synergistic effects on pheromone detection at the OR level.

Ionotropic receptors have been traditionally associated with acid and amine detection. Our analysis identified at least 14 putative antennal IRs (Fig. 6). Considering that fermentation products seem to be essential for aggregation and oviposition of palm weevils, it is expected that some of such IRs may be tuned to compounds of yeast and bacterial origin, especially those more closely related to deorphanized *D. melanogaster* IRs receptors such as IR75a and IR75d, tuned to acetic acid and pyrrolidine, respectively^77^. A comparison between APW and RPW IRs revealed that most APW IRs have a direct orthologous IR from RPW. Within the antennal IR, however, subfamily, there are several cases of divergence between IRs from the two species. Whether these IRs perform different functions in each palm weevil species is unknown since it is challenging to assume any particular function for the IRs identified.

This comprehensive analysis of chemosensory related proteins provides a fundamental resource to better understand the APW chemoreception and serve as a basis for comparative studies with the APW Asian counterpart, the RPW. We highlighted differences and commonalities between these closely related species of weevils that share palms as their hosts but occupy different, non-overlapping areas globally. Further functional studies will better understand how olfactory gene evolution correlates with their host range and ethology^78^. The advent of higher computational power and machine-learning technologies will increase the capacity to find new ligands when the receptors are known^79^. Detecting conserved genetic traits and their functional bases emerges as an essential tool to predict and identify pheromone component and kairomone responses and, consequently, deepen our knowledge of pest insect chemical senses that could improve their management.

## Data availability

All sequence reads were submitted to the SRA of NCBI under the accession numbers: SRR12450122 – APW male and SRR12450123 – APW female. This Transcriptome Shotgun Assembly project has been deposited at DDBJ/EMBL/GenBank under the accession GIUZ00000000. The RpalOR1, RpalOBP4_1 and RpalOBP10 sequences reported in this paper have been deposited in the GenBank database (Accession Nos. MT887347-MT887349).

## Supplementary Information

Supplementary Information 1.

Supplementary Information 2.
