# Author Correction: Antennal transcriptome sequencing and identification of candidate chemoreceptor proteins from an invasive pest, the American palm weevil, *Rhynchophorus palmarum*

**DOI:** 10.1038/s41598-021-96402-8

**Published:** 2021-08-19

**Authors:** Francisco Gonzalez, Jibin Johny, William B. Walker, Qingtian Guan, Sara Mfarrej, Jernej Jakše, Nicolas Montagné, Emmanuelle Jacquin‑Joly, Abdulaziz S. Alqarni, Mohammed Ali Al‑Saleh, Arnab Pain, Binu Antony

**Affiliations:** 1 Chair of Date Palm Research, Center for Chemical Ecology and Functional Genomics, Department of Plant Protection, College of Food and Agricultural Sciences, King Saud University, Riyadh, 11451 Saudi Arabia; 2 Department of Research and Development, ChemTica Internacional S.A., Santo Domingo, Heredia, Costa Rica; 3 Department To Plant Protection Biology, Swedish University of Agricultural Sciences, Alnarp, Sweden; 4 Biotechnical Faculty, Agronomy Department, University of Ljubljana, 1000 Ljubljana, Slovenia; 5 INRAE, Sorbonne Université, CNRS, IRD, UPEC, Université de Paris, Institute of Ecology and Environmental Sciences of Paris, iEES-Paris, 78000 Versailles, France; 6 BESE Division, King Abdullah University of Science and Technology (KAUST), Thuwal, Jeddah, 23955‑6900 Saudi Arabia

Correction to: *Scientific Reports* https://doi.org/10.1038/s41598-021-87348-y, published online 15 April 2021

The original version of this Article contained an error in the spelling of the author Abdulaziz S. Alqarni which was incorrectly given as Abdulaziz A. Alqarni.

As a result, the Author Contributions section now reads:

“B.A., E.J.J., and A.P. conceived the study, acquired the grant, and participated in its design and coordination. F.G. and W.W. participated in the A.P.W. collection. Q.G., S.M., J.J., B.A., and A.P. carried out Illumina sequencing. Je.J., B.A., A.S.A. and M.A.L. carried out the trimming, de novo assembly, quality control analysis, the Blast2GO analysis, and local BLASTx searches. F.G., W.W., J.J., N.M., and B.A. performed phylogenetic analysis. F.G., B.A., J.J., and W.W. wrote the paper with contributions from N.M., E.J.J., Je.J., and A.P., and all authors have read and approved the final manuscript.”

The original Article and accompanying Supplementary Information file have been corrected.

