# Supplementary material for: Antennal transcriptome sequencing and identification of candidate chemoreceptor proteins from an invasive pest, the American palm weevil, Rhynchophorus palmarum
Source: Sci Rep. 2021 Apr 15;11:8334. doi: 10.1038/s41598-021-87348-y (PMC8050089; doi:10.1038/s41598-021-87348-y)

***Scientific Reports***

**Supplementary Information for**

**Antennal transcriptome sequencing and identification of candidate chemoreceptor proteins from an invasive pest, the American palm weevil, Rhynchophorus palmarum**

**Francisco Gonzalez^1,2, #^, Jibin Johny^1, #^, William B. Walker III^3, #^, Qingtian Guan^6^, Sara Mfarrej^6^, Jernej Jakše^4^, Nicolas Montagné^5^, Emmanuelle Jacquin-Joly^5^, Abdulaziz S. Alqarni^1^, Mohammed Ali Al-Saleh^1^, Arnab Pain^6^ and Binu Antony^1,*,#^**

^1^King Saud University, Chair of Date Palm Research, Center for Chemical Ecology and Functional Genomics, Department of Plant Protection, College of Food and Agricultural Sciences, Riyadh 11451, Saudi Arabia.

^2^Department of Research and Development, ChemTica Internacional S.A., Santo Domingo, Heredia, Costa Rica.

^3^Department to Plant Protection Biology, Swedish University of Agricultural Sciences, Alnarp, Sweden.

^4^University of Ljubljana, Biotechnical Faculty, Agronomy Department, SI-1000 Ljubljana, Slovenia

^5^INRAE, Sorbonne Université, CNRS, IRD, UPEC, Université de Paris, Institute of Ecology and Environmental Sciences of Paris, iEES-Paris, F-78000 Versailles, France

^6^King Abdullah University of Science and Technology (KAUST), BESE Division, Thuwal,

Jeddah 23955-6900, Saudi Arabia.

**To whom correspondence may be addressed: E-mail:* [*bantony@ksu.edu.sa*](mailto:bantony@ksu.edu.sa)

**This PDF file includes:**

Supplementary Figure S1a.

Supplementary Figure S1b.

Supplementary Figure S2.

Supplementary Figure S3.

Supplementary Figure S4.

**Other supplementary materials for this manuscript include the following:**

Supplementary Table S1 to S7.

**Supplementary Figures**

**Figure S1a.** *R. palmarum* male transcriptome gene ontology analysis (Blast2GO) results. Graphs showing total sequences annotated, data distribution, database distribution, enzyme code distribution, top blast hit species distribution and GO distribution at three functional levels as Biological Process (BP), Molecular Functions (MF) and Cellular Component (CC).


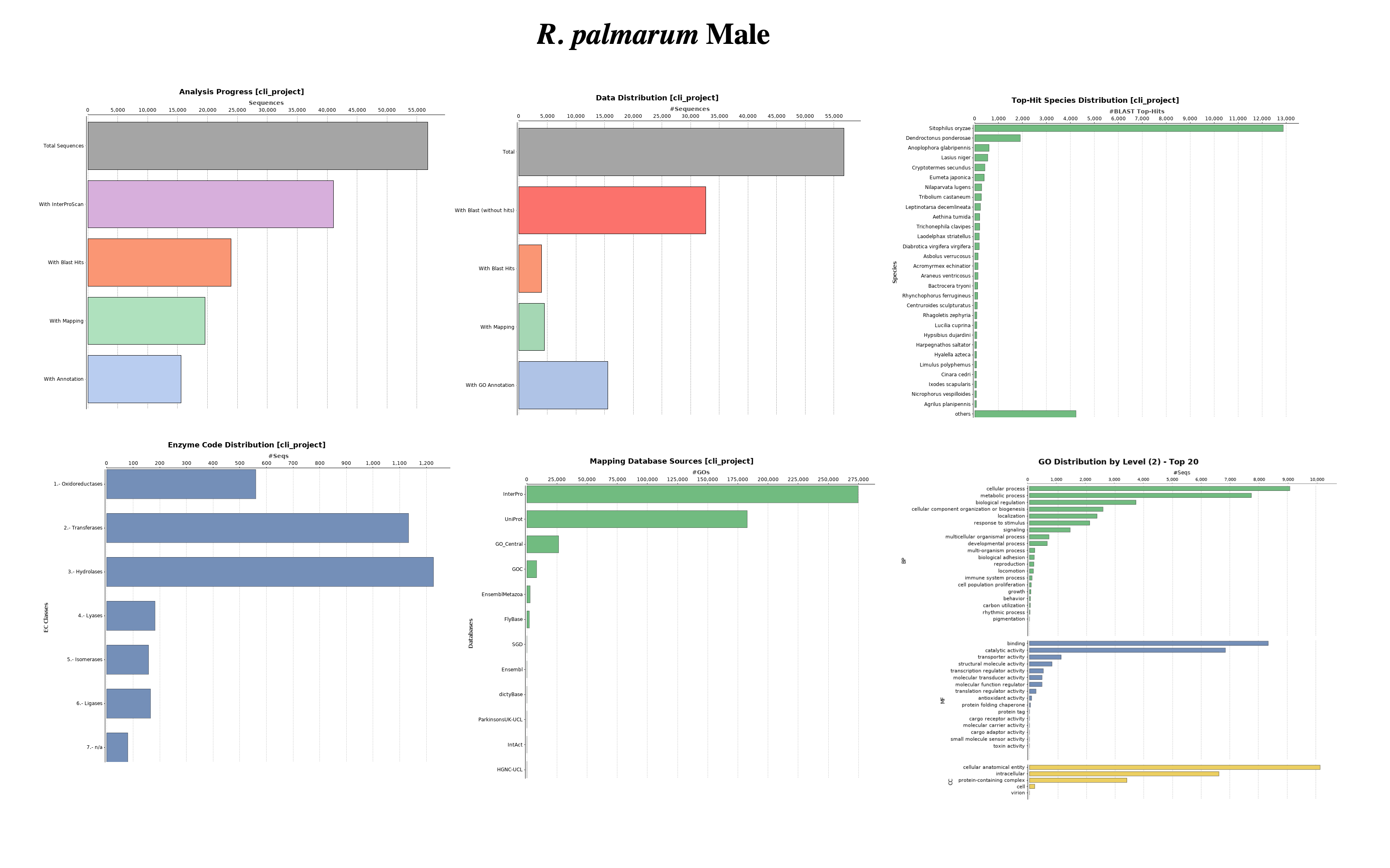


**Figure S1b.** *R. palmarum* female transcriptome gene ontology analysis (Blast2GO) results. Graphs showing total sequences annotated, data distribution, database distribution, enzyme code distribution, top blast hit species distribution and GO distribution at three functional levels as Biological Process (BP), Molecular Functions (MF) and Cellular Component (CC).


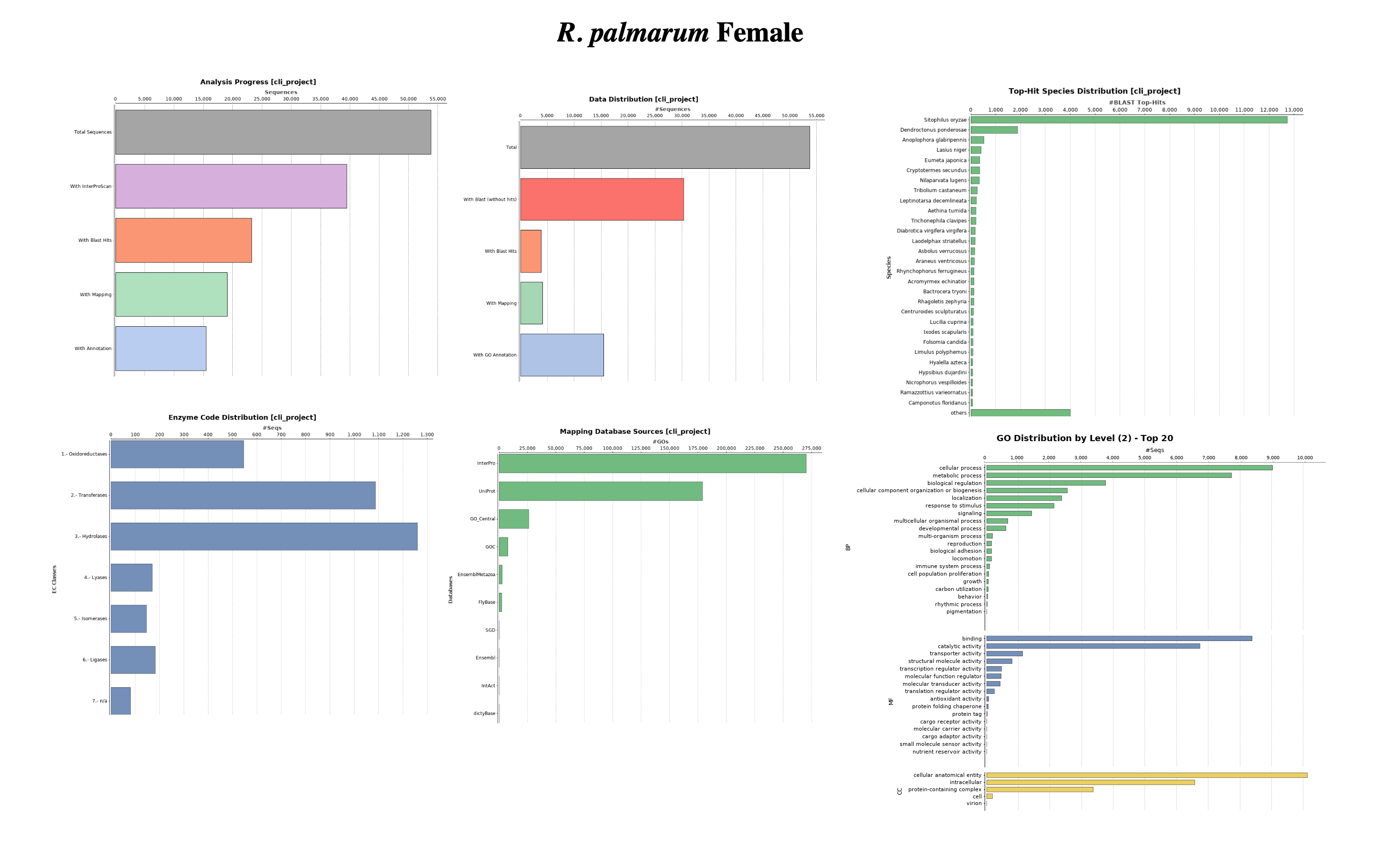


**Figure S2.** Multiple sequence alignment of predicted RpalOBPs. The figure only shows partial sequences. Dashed boxes show coinciding conserved cysteines among the sequences. They were aligned with MAFFT.


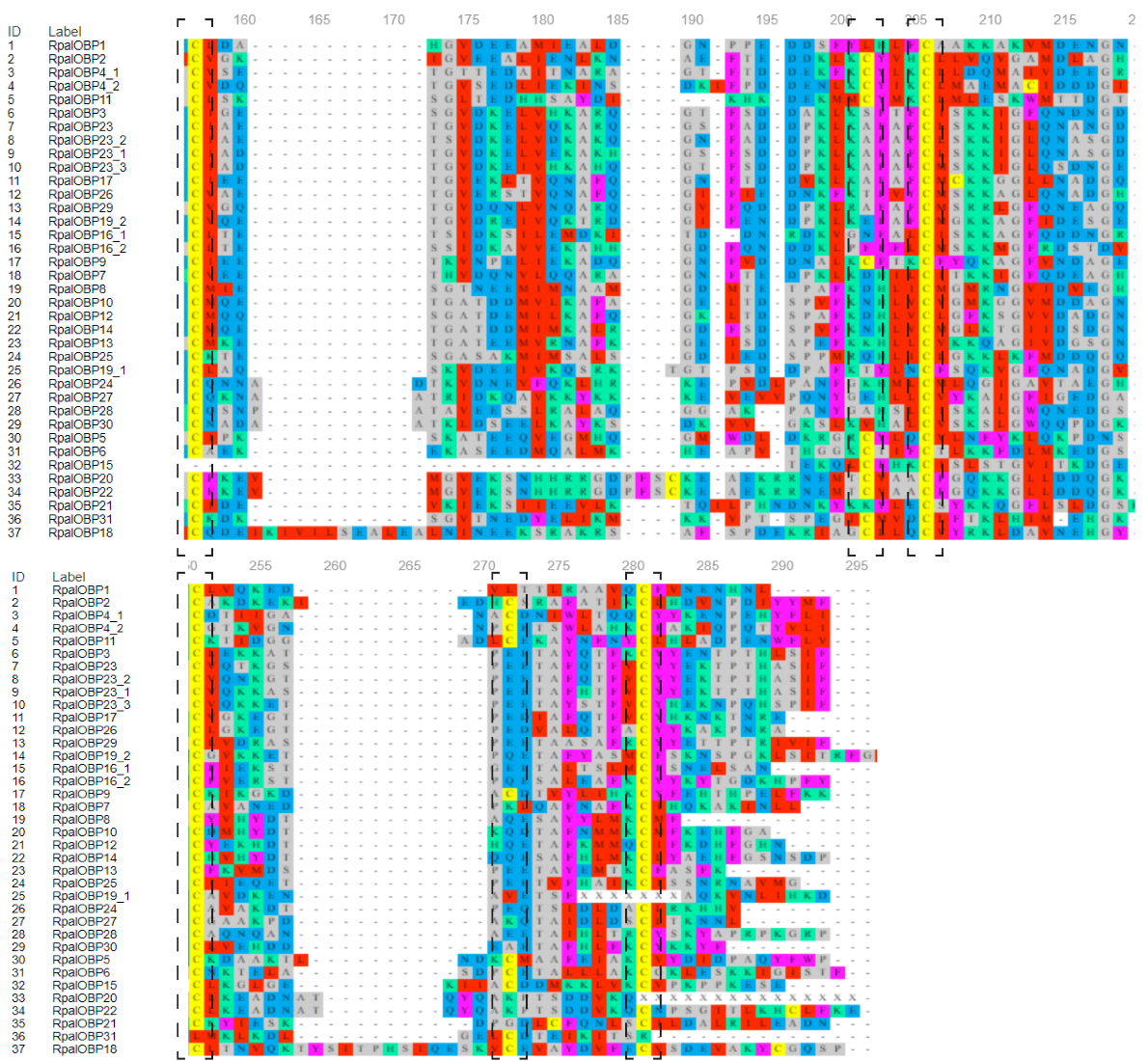


**Figure S3.** Multiple sequence alignment of predicted RpalCSPs. The figure only shows a partial sequence with dashed boxes detailing coinciding conserved cysteines among the sequences. They were aligned with MAFFT.


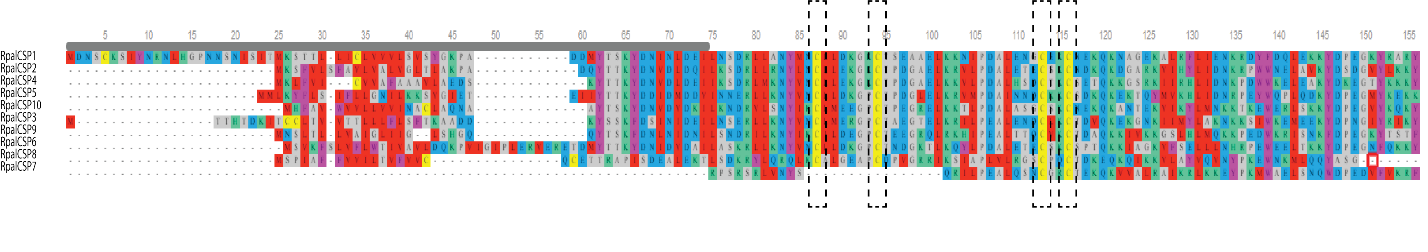


**Figure S4.** Multiple sequence alignment of predicted RpalGRs. The figure only shows a partial sequence with a dashed box detailing coinciding pattern among the sequences. They were aligned with MAFFT.


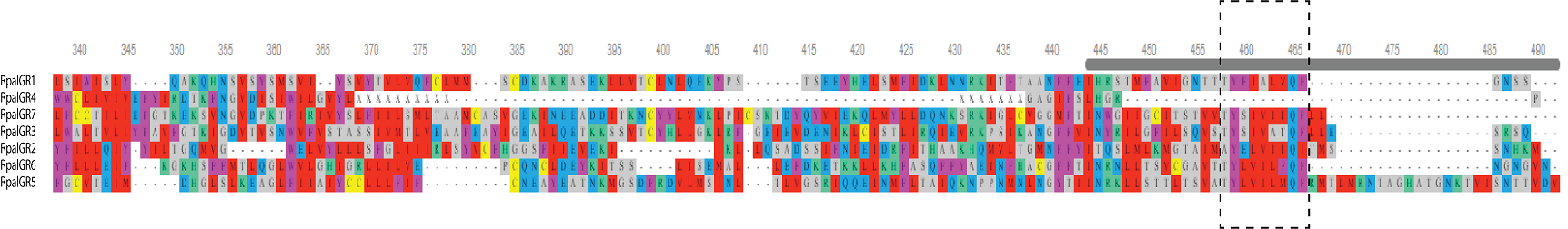

Supplement: Supplementary file 1 — Supplementary Information 1. [file 41598_2021_87348_MOESM1_ESM.docx]
